# Mechanical stability study of three techniques used in the fixation of transverse and oblique metaphyseal-diaphyseal junction fractures of the distal humerus in children: a finite element analysis

**DOI:** 10.1186/s13018-020-1564-4

**Published:** 2020-01-31

**Authors:** Chuang Liu, Allieu Kamara, Tianjing Liu, Yunhui Yan, Enbo Wang

**Affiliations:** 1 State Key Laboratory of Mechanical Behavior and System Safety of Traffic Engineering Structures, Shijiazhuang Tiedao University, Shijiazhuang, 050000 Hebei People’s Republic of China; 2 Department of Pediatric Orthopedics, Shengjing Hospital of China Medical University, Shenyang, 110004 Liaoning People’s Republic of China; 3 School of Mechanical Engineering & Automation, Northeastern University, Shenyang, 110819 Liaoning People’s Republic of China

**Keywords:** Metaphyseal-diaphyseal junction fracture, Supracondylar humerus fracture, Pinning fixation, External fixator, Elastic stable intramedullary nails, Biomechanical study, Finite element analysis

## Abstract

**Background:**

Management of distal humerus metaphyseal-diaphyseal junction (MDJ) region fractures can be very challenging mainly because of the higher location and characteristics of the fracture lines. Loss of reduction is relatively higher in MDJ fractures treated with classical supracondylar humerus fractures (SHFs) fixation techniques.

**Methods:**

Three different fracture patterns including transverse, medial oblique and lateral oblique fractures were computationally simulated in the coronal plane in the distal MDJ region of a pediatric humerus and fixated with Kirschner Wires (K-wires), elastic stable intramedullary nails (ESIN), and lateral external fixation system (EF). Stiffness values in flexion, extension, valgus, varus, internal, and external rotations for each fixation technique were calculated.

**Results:**

In the transverse fracture model, 3C (1-medial, 2-lateral K-wires) had the best stiffness in flexion, varus, internal, and external rotations, while 3L (3-divergent lateral K-wires) was the most stable in extension and valgus. In the medial oblique fracture model, EF had the best stiffness in flexion, extension, valgus, and varus loadings, while the best stiffness in internal and external rotations was generated by 3MC (2-medial, 1-lateral K-wires). In the lateral oblique fracture model, 3C (1-medial, 2-lateral K-wires) had the best stiffness in flexion and internal and external rotations, while ESIN had the best stiffness in extension and valgus and varus loadings.

**Conclusion:**

The best stability against translational forces in lateral oblique, medial oblique, and transverse MDJ fractures would be provided by ESIN, EF, and K-wires, respectively. K-wires are however superior to both ESIN and EF in stabilizing all three fracture types against torsional forces, with both 2-crossed and 3-crossed K-wires having comparable stability. Depending on the fracture pattern, a 3-crossed configuration with either 2-divergent lateral and 1-medial K-wires or 2-medial and 1-lateral K-wires may offer the best stability.

## Background

Distal humerus metaphyseal-diaphyseal junction (MDJ) region fracture in children is a complex fracture which requires accurate management by a trained pediatric orthopedic surgeon. Management is mainly directed towards restoring bone healing as well as managing fracture-related complications, in order to restore a cosmetically normal and functional limb to the child. However, complications still occur after using the current treatment modalities available for typical supracondylar humerus fractures (SHFs). One of the most common complications following management of displaced SHF is loss of reduction. The incidence of loss of reduction with the gold standard percutaneous pinning technique alone has been reported to be as high as 18%, and most of the cases would require secondary management [1] or may develop into unwanted complications, which may pose a significant burden on both patient and caregiver.

Pin configuration and pin spread along the fracture line among other factors have been said to be associated with loss of reduction in the management of SHFs [2–5]. To effectively manage distal humerus MDJ fractures, special attention must be paid to the characteristics of the fracture lines. Difficulty in achieving and maintaining anatomic alignment can be attributed to the fracture line patterns, especially when closed reduction and percutaneous pinning is to be utilized. Because of the higher location of the fracture line and the variability in characteristics of the fracture line, coupled with the angulation of the metaphyseal flare relative to the humeral shaft, reduction and pinning fixation of these fractures may be technically challenging. Adequate stability cannot be guaranteed even if fixation is achieved, due to the small cortical bone of the proximal fragment available for pin purchase. These “supra-olecranon fossa” fractures are shown to have a higher incidence of post-op complications than the classical “trans-olecranon” types [6, 7]. In the search for more stable techniques for these challenging and unstable fractures, other techniques such as lateral external fixators (EF) and elastic stable intramedullary nails (ESIN) have been proposed as alternative fixation methods, and most have yielded satisfactory outcomes [8–11]. However, a comparative study of these techniques in these fractures with varying characteristics, using a pediatric humerus model is still lacking in the literature.

In a previous biomechanical study, we compared the stability of Kirschner wires (K-wires), ESIN and EF in various heights distal humerus MDJ fractures using composite bone models [12]. Fractures located in the upper half of the distal MDJ region were found to be more stable with ESIN, while fractures located in the lower half were more stable with K-wires. However, only transverse type fractures were tested in that study. Moreover, adult-size bones instead of pediatric-size ones were used. Composite bones and other synthetic bones that have been used in most biomechanical tests differ structurally and mechanically from that of pediatric bone and therefore cannot completely mimic the physiologic parameters of a pediatric bone. In this current study, we compared the mechanical stability of K-wires, ESIN, and EF, in distal humeral MDJ fractures of various characteristics, using computationally modeled pediatric humerus and finite element (FE) analysis, in order to get a deeper and better understanding of the biomechanical performance of the three fixation techniques.

## Materials and methods

### Fracture and fixation simulations

**Fig. 1:**
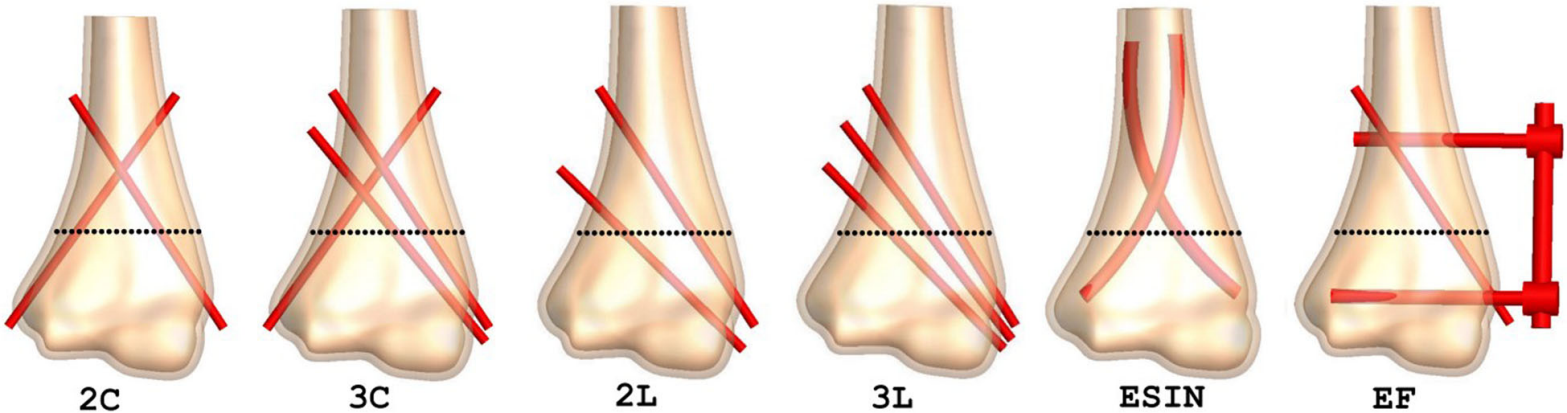
Fixation simulations for the transverse fracture model. 2C, two-crossed pins (1-medial and 1-lateral K-wires); 3C, three-crossed pins (1-medial and 2-lateral K-wires); 2L, two-lateral pins (2-divergent lateral K-wires); 3L, three-lateral pins (3-divergent lateral K-wires); ESIN, elastic stable intramedullary nails; EF, lateral external fixation system

**Fig. 2:**
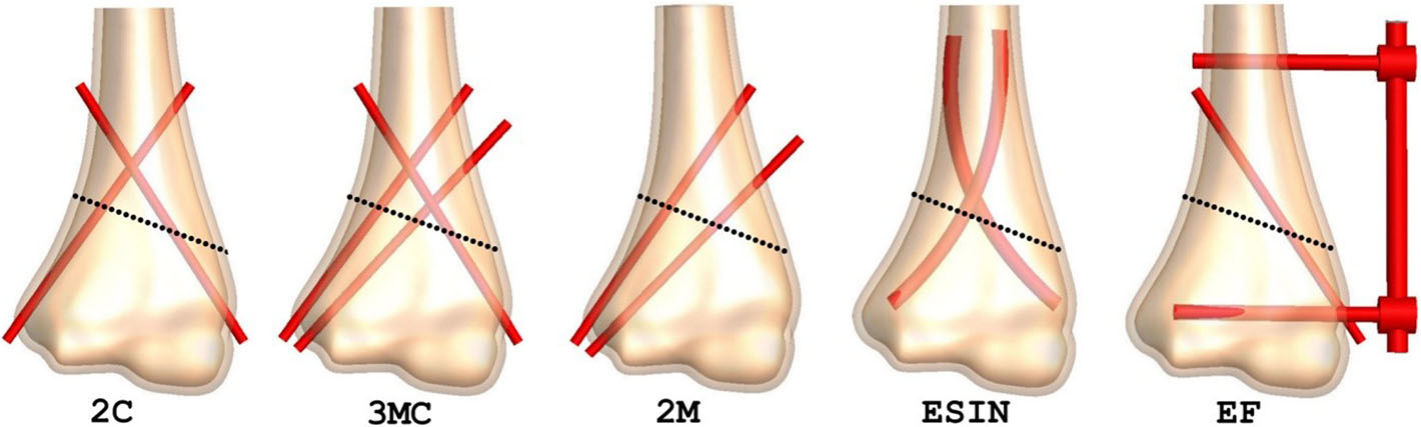
Fixation simulations for the medial oblique fracture model. 2C, two-crossed pins (1-medial and 1-lateral K-wires); 3MC, three-crossed pins (2-medial and 1-lateral K-wires); 2M, two-medial pins (2-divergent medial K-wires); ESIN, elastic stable intramedullary nails; EF, lateral external fixation system

**Fig. 3:**
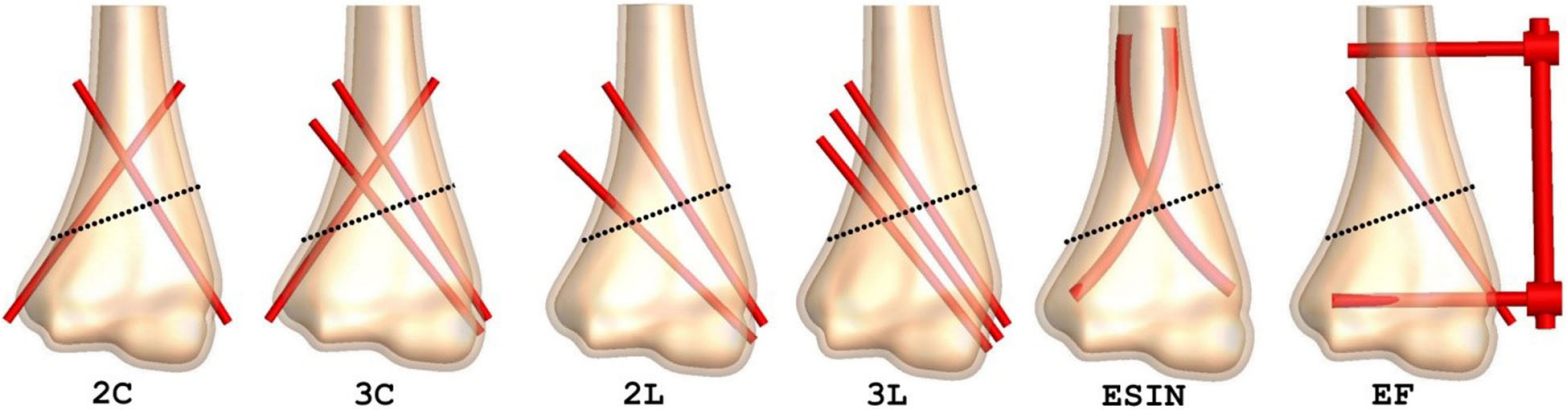
Fixation simulations for the lateral oblique fracture model. 2C, two-crossed pins (1-medial and 1-lateral K-wires); 3C, three-crossed pins (1-medial and 2-lateral K-wires); 2L, two-lateral pins (2-divergent lateral K-wires); 3L, three-lateral pins (3- divergent lateral K-wires); ESIN, elastic stable intramedullary nails; EF, lateral external fixation system

After approval from our institutional review boards, a 3D-CT scan data of a 10-year-old boy in Digital Imaging and Communications in Medicine (DICOM) format was obtained for this study. The boy was evaluated for an occult fracture to the elbow after a minor trauma but had no evidence of that on the scan. The CT scan had a slice thickness of 0.5 mm (Brilliance 64ME; Philips, Eindhoven, The Netherlands). The data were imported into Simpleware 6.0 (Synopsis Inc., CA, USA) for segmentation and 3D reconstruction. A three-dimensional model of the distal humerus comprising of a cancellous bone internally and a cortical bone layer externally was developed from the CT images. This reconstructed surface humerus model was then imported into SolidWorks 2016 edition (Dassautt Systemes-Simula, France) in international graphic interactive standard (IGES) format, for solid model generation and fracture and fixation simulations. Before fracture simulation, the distal MDJ region was determined as previously described [7, 12]. Three different distal humerus MDJ fracture patterns including transverse, medial oblique, and lateral oblique fractures were simulated in the coronal plane. According to Bahk et al., fractures with a coronal obliquity of 10 or more or fractures with a sagittal obliquity of 20° or more are associated with a significant difference in additional characteristics, treatment, and outcomes [6]. The transverse fracture in our model, therefore, had a 20° sagittal obliquity, while the oblique fractures had coronal obliquities of 20°. The medial oblique fracture started at the lateral edge above the olecranon fossa and exited proximally medially, while the lateral oblique fracture started at the medial edge above the olecranon fossa and exited proximally laterally. The fractures were then fixated with K-wires, lateral external fixation system (EF), and elastic stable intramedullary nails (ESIN) as previously described [8, 12]. All K-wires had a diameter of 2.0 mm. ESIN comprised of two 3.0 mm titanium nails and EF comprised of two 3.0 mm threaded half-pins, one 4.0 mm stainless steel connecting rod, two connectors, and one free lateral-entry K-wires. All lateral-entry K-wires were direct entry pins in divergent configuration. A total of 17 fixation models were generated for the FE analysis (Figs. 1, 2, and 3).

### Finite element model preparation and simulations

The generated fixated models were imported into Hypermesh 14 (Altair Engineering, MI, USA) for meshing. After meshing of all the models, they were finally imported into Abaqus 6.14 (Dassault Systemes-Simula, France) for the FE analysis. The number of elements and nodes for bone and hardware, as well as their material properties, are shown in Table 1 [13]. As the stiffness of hardware is much larger than bone tissue, embedding constraints were used between elements of the hardware in order for hardware to be embedded into the bones. The interactions among the humerus, pins, and rods were defined as binding constraints. The proximal end of the humerus was completely restrained, and a control point was selected on the distal surface of the joint line along the humeral midline. A magnitude of 30 N concentrated force and a 1.5 Nm moment were applied at the control point along the *X* − *X*, *Y*, − *Y*, *Z,* and − *Z* axes, respectively, to assess for the stability of the fixation models.

**Table 1:** Material properties, number of elements, and nodes of the finite element model

Material	Element type	Young’s modulus (MPa)	Poison’s ratio	Number of element	Number of nodes
Cortical bone	C3D4	16,000	0.3	56,678	15,617
Cancellous bone	C3D4	150	0.3	48,736	10,906
K-wire	C3D8R	200,000	0.33	456	696
ESIN	C3D8R	110,000	0.33	1872	2618
External fixator	C3D8R and C3D4	200,000	0.33	5359	3837

### Determination of model stiffness

FE analysis of the fixated humeral model was divided into translational force and torque. The translation force was applied in the four loading directions of flexion, extension, valgus, and varus. The stiffness of the model K_F_ in the four loading directions was derived from the formula: *K*_*F*_ = *F*/*X*, where *F* denotes the applied force in Newtons (N), *X* is the actual deformation of the model in millimeters in the post FE analysis. The displacement *X* produced by force *F* was obtained from the displacement nephograms of Abaqus. This was calculated by determining the distance between chosen representative nodes before and after deformation. Similarly, stiffness of the model *K*_*M*_ of the applied moment *M* in internal and external rotations were derived from the formula: *K*_*M*_ = *M*/*θ*, where *M* is the moment in Newton millimeters (Nmm) and *θ* is the angular variable quantity in degrees (°) that was achieved by the bone model in the post-analytic results.

## Results

### Stiffness of the fixation models

Tables 2, 3, and 4 show the calculated stiffness values for the fixation models.

**Table 2:** Stiffness values for the transverse fracture model fixation simulation

Fixation	Flexion	Extension	Valgus	Varus	Internal rotation	External rotation
2C	23.3100	5.1800	14.7275	14.8157	172.4983	172.9134
3C	33.6587	11.2196	21.7328	33.6587	187.7382	189.1732
2L	19.3562	10. 6235	21.6709	27.5811	93.8844	93.8896
3L	29.3542	14.6771	23.7567	30.2358	101.6560	101.2576
EF	28.1955	6.2657	11.4714	14.6000	85.1572	84.5978
ESIN	22.1607	12.3115	14.9275	14.0977	149.6425	149.4406

Data of flexion, extension, valgus, and varus loadings are in N/mm, while data of internal and external rotations are in Nmm/°. *2C* two-crossed pins (1-medial and 1-lateral K-wires), *3C* three-crossed pins (1-medial and 2-lateral K-wires), *2L* 2-lateral K-wires, *3L* 3-lateral K-wires, *EF* lateral external fixator, *ESIN* elastic stable intramedullary nails

**Table 3:** Stiffness values for the medial oblique fracture model fixation simulation

Fixation	Flexion	Extension	Valgus	Varus	Internal rotation	External rotation
2C	25.7397	25.651	14. 7933	16.5231	148.796	217.5591
3MC	48.9950	31.0508	17.7905	21.2805	173.7579	257.8124
2M	28.9209	30.452	14. 8697	19.1903	82.7283	89.3163
EF	52.0703	62.5383	28.8646	21.6523	61.6056	57.4632
ESIN	22.5832	22.5884	11.9323	15.3845	139.5170	134.9637

Data of flexion, extension, valgus, and varus loadings are in N/mm, while data of internal and external rotations are in Nmm/°. *2C* two-crossed pins (1-medial and 1-lateral K-wires), *3C* three-crossed pins (1-medial and 2-lateral K-wires), *2M* 2-medial K-wires, *EF* lateral external fixator, *ESIN* elastic stable intramedullary nails

**Table 4:** Stiffness values for the lateral oblique fracture model fixation simulation

Fixation	Flexion	Extension	Valgus	Varus	Internal rotation	External rotation
2C	108.1910	113.6450	193.1430	169.0100	183.8870	184.3140
3C	125.8300	129.5290	225.7960	173.0010	221.9005	238.4727
2L	102.0450	81.5110	189.2530	133.0720	89.6237	91.0009
3L	110.5610	82.4640	202.7530	146.5370	95.3620	98.2150
EF	61.9450	51.2210	124.6650	171.7150	62.5179	68.1112
ESIN	121.9760	139.6650	236.4530	204.6500	154.9460	146.6370

Data of flexion, extension, valgus, and varus loadings are in N/mm, while data of internal and external rotations are in Nmm/°. *2C* two-crossed pins (1-medial and 1-lateral K-wires), *3C* three-crossed pins (1-medial and 2-lateral K-wires), *2L* 2-lateral K-wires, *3L* 3-lateral K-wires, *EF* lateral external fixator, *ESIN* elastic stable intramedullary nails

In the transverse fracture model, the 3C (1-medial, 2-lateral K-wires) configuration had the best stiffness in flexion, varus, internal, and external rotations, while the 3L (3-divergent lateral K-wires) configuration was the most stable in extension and valgus (Table 2). EF was the least stable in all other loading directions, except in flexion and extension where 2L (2-divergent lateral K-wires) and 2C (1-medial, 1-lateral K-wires) were respectively weakest.

In the medial oblique fracture model, EF had the best stiffness in flexion, extension, valgus, and varus loadings, while the best stiffness in internal and external rotations was generated by 3MC (2-medial, 1-lateral K-wires). The second best configurations were 2M (2-medial K-wires) in translational loadings and 2C (1-medial, 1-lateral K-wires) in torsional loadings. ESIN had the least stiffness in translational loadings, while EF was the weakest in torsional loadings (Table 3).

In the lateral oblique fracture model, 3C (1-medial, 2-lateral K-wires) had the best stiffness in flexion, internal, and external rotations, while ESIN had the best stiffness in extension, valgus, and varus loadings. The second best stiffness in torsional tests was generated by 2C (1-medial, 1-lateral K-wires). Overall, EF had the least stiffness values, except in varus where 2L (2-divergent lateral K-wires) was the weakest (Table 4).

## Discussion

Management of distal humerus MDJ fractures can be very challenging not only because of the unique anatomy of the distal humerus but mainly because of the higher location and characteristics of the fracture lines. In this study, we established a pediatric humerus model and compared the stability of common fixation techniques across three different fracture patterns in the distal MDJ region, using a combination of innovative software packages. These kinds of software allow us to simulate biomechanical studies and provide us with informative data which cannot easily be obtained through traditional biomechanical studies. When compared to traditional biomechanical studies, this study is also novel in a way that, the same pediatric humeral bone model is used for all simulation and analysis, and the fixation simulation for the same technique is homogenous across the different fracture models, thereby obtaining results that are much more accurate and reliable.

In a previous biomechanical study conducted with composite bone models [12], K-wires were found to be superior to both ESIN and EF in stabilizing transverse fractures that are located in the lower MDJ region. Other fracture patterns such as fractures with coronal obliquity were however not investigated in that study. In this study, in addition to the transverse fracture, we also investigated two different fractures with coronal obliquity. The characteristics of the fracture lines were the main determining and limiting factors in our choice of K-wire configurations. For transverse and lateral oblique fractures, it was easily possible to place 2 or 3 K-wires laterally. However, for the medial oblique fractures, it was impossible to place more than one lateral K-wire. Two K-wires were therefore placed medially, to give the 2M and 3MC configurations.

In this current study, K-wire pinning fixations were in most cases also found to be superior to both ESIN and EF in the transverse and oblique fracture models. In the transverse fracture models, the results showed 3-divergent lateral K-wires (3L) to have the most resistance against translational forces, while 3-crossed K-wires were the best in resisting torsional forces. In oblique MDJ fractures, however, K-wires were only superior in torsional loadings, with 3-crossed K-wires possessing the best anti-torsional ability, followed by 2-crossed K-wires. The 2-medial K-wires used in the medial oblique fracture model were found to be more stable than 2-crossed K-wires against translational forces but were inferior to 2-crossed pins against torsional forces. Wang et al. [14] found no statistical difference between 2-medial pins and 2-crossed pins configurations in their low medial oblique fracture model. Their 2-crossed pins configuration was found to provide the greatest stability in varus, internal, and external rotations. Apart from the different humeral models used, the location and angle of the fracture lines in their study differed from ours, which could all be responsible for these different results.

Numerous studies have compared the stabilization effect of crossed and lateral-only pinning configurations for typical SHFs. Lee et al. [15] found comparable stability between crossed and divergent lateral K-wires, except under torsional loadings, where the crossed configuration offered better stability. Feng and co-authors [16] showed that 2 to 3 lateral K-wires were superior under most loading conditions. Lamdan R et al. in their finite element analytic study on pediatric fractures performed with composite bone model concluded that under normal bone-implant interface conditions, 2-divergent lateral K-Wires configuration offers satisfactory mechanical stability and may be the preferred choice of SHF fixation [17]. They, however, suggested 3-divergent lateral K-wires as an alternative to crossed K-wires. In our study, 3-divergent lateral pinning configuration was only found to be more stable than 2-crossed K-wires in flexion and valgus loadings in the lateral oblique fracture but was more stable than both 2 and 3-crossed K-wire configurations in extension and valgus in the transverse fracture. It was, however, weaker than the two configurations in torsional loadings in both fracture models. This implies that, despite their superiority against translational forces, lateral pins still remain inferior in resisting torsional forces especially in coronal oblique fractures, irrespective of the number of K-wires used.

K-wire number was directly related to the stiffness of pinning configurations to some degree. In translational loadings, 3-crossed K-wires were overall 42% stiffer than 2-crossed K-wires in transverse fractures but were only 13% stiffer in medial oblique fractures and 11% stiffer in lateral oblique fractures. In torsional loadings, the 3-crossed K-wires were 28% stiffer than 2-crossed K-wires in the medial oblique fractures, 20% stiffer in the lateral oblique fractures, and only 8.4% stiffer in the transverse fractures. The results indicate that three-crossed K-wires in either 2-medial plus 1-lateral or 1-medial plus 2-lateral K-wires configuration would confer better stability against torsional forces in fractures that are more transverse in the MDJ region than 2-crossed K-wires alone, and that both 3-crossed and 2-crossed K-wires can offer sufficient but comparable stability against torsional forces in these fractures. As for the lateral pinning configurations, three-divergent lateral K-wires were only 19% stiffer than two-divergent lateral K-wires against translational forces in the transverse fractures and 7% stiffer in the lateral oblique fractures. In torsional loadings, the difference between both configurations was not too obvious. The 3-divergent lateral K-wires were only 8% stiffer than 2-divergent lateral K-wires in the transverse fractures and 7% stiffer in the lateral oblique fractures. A study conducted by Jaeblon and colleagues however discovered significant greater torsional stiffness with 3-divergent lateral K-wires than 2-divergent lateral K-wires in their high transverse fracture model [18]. However, they found no significant difference between the pinning configurations in coronal or sagittal stiffness. Divergence of the two results could probably be explained by the variations of humeral models used.

ESIN, which has been shown to possess superior stabilizing capability than K-wires in higher MDJ fractures [12], performed poorly in our transverse and medial fracture models. It was found to be the weakest against translational forces in the medial oblique fracture and remained weaker against torsional forces in the lateral oblique fracture model. In the lateral oblique fractures, however, performance in extension and sagittal loadings was second to none. Even though perfect alignment can be achieved with ESIN, stability may be compromised if the nails cross near the fracture site [12]. The relatively higher location of the fracture lines in the distal humeral MDJ region caused the nails to cross closer to the fracture sites, thereby bringing the center of rotation of the nails closer to the fracture line, rendering the technique inferior. This study further demonstrates that the more oblique the fracture line is in the coronal plane, the closer the crossing point of nails to the fracture line would be, and therefore the weaker the technique can be against torsional forces. It was also observed that, if the fracture site falls distal to the crossing point, stability against translational forces can be compromised. This is mainly because the three-point fragment fixation principle of ESIN cannot be obeyed in these cases [12, 19]. The distal fracture fragments that are located distally to the crossing point would, therefore, be loosely held by the nails, thereby affecting their stability.

In as much as multiple clinical studies have shown satisfactory results with EF [8, 20] overall, the technique was found to be the weakest in two of our fracture models. Hohloch et al. in an earlier study found EF with ulnarly placed K-wire to be more stable than crossed pinning in internal rotation [21, 22] and therefore recommended insertion of an additional ulnarly anti-rotation K-wire instead of a radial one in cases of pediatric SHFs when an external fixator is used for osteosynthesis, because this may reduce secondary displacement of the distal fragment. However, because ulna K-wires has the risk of injuring the ulnar nerve, its use is mostly avoided by many surgeons. In our medial oblique fractures, however, the EF system with a radially placed K-wire was found to be the most stable against translational forces among all the techniques but however performed poorly in torsional loadings in the fracture models. A probable explanation for the high performance of EF in the medial oblique fracture model is that, the radial K-wire which crossed the fracture line at the more distal end of the K-wire had its center of force concentration and rotation located proximally above the fracture line, yielding the technique more stability. On the other hand, the center of force concentration and rotation in the other fractures was more close to the fracture lines, thereby making them unstable with external forces. Our study, however, differed from theirs in humerus model, fracture location, and patterns.

Despite the novelty of this study, some limitations need to be acknowledged. The humerus model was based on the reconstructed 3D humerus model from the CT images. During the modeling process, we needed to do the necessary simplifications of the model, in order to avoid errors in meshing and analysis. The model also lacked other child characteristic structures like the distal cartilage, which may not completely represent the actual pediatric humerus. Moreover, the material parameters of the biomechanical simulation were mostly of ideal homogeneous materials, which may be different from that of true biological tissue and biomechanical model. Furthermore, FE solution in itself uses approximate calculations instead of actual biological models, so the calculations of the final results may differ from that of actual results. However, because the same reconstructed humerus bone model was used for all fracture and fixation simulations, and since fixation simulation for the same technique was homogenous across the three fracture models, the results obtained would not have been much influenced by the these limitations, since our ultimate goal was to compare fixation techniques across the three fracture models.

## Conclusions

This study demonstrates that FE analysis is an effective and accurate way to simulate biomechanical studies, which can serve as an alternative to the more time-consuming traditional biomechanical studies, as it obtains accurate results in a shorter period of time. From a biomechanical perspective, the best stability against translational forces in the lateral oblique, medial oblique and transverse MDJ fractures would be provided by ESIN, EF, and K-wires, respectively. K-wires are however superior to both ESIN and EF in stabilizing all three fracture types against torsional forces, with both two-crossed and three-crossed K-wires having comparable stability. Depending on the fracture line, three-crossed configuration with either two-divergent lateral and one-medial K-wires or two-medial and one lateral K-wires may offer the best stability. Clinical investigations are however necessary to further verify these findings.

## Data Availability

Corresponding author Enbo Wang can be contacted to request the raw data.

## Abbreviations

2L: Two-lateral pins (2-divergent lateral K-wires)
2M: Two-medial pins (2-divergent medial K-wires)
2C: Two-crossed pins (1-medial and 1-lateral K-wires)
3L: Three-lateral pins (3-divergent lateral K-wires)
3C: Three-crossed pins (1-medial and 2-lateral K-wires)
3MC: Three-crossed pins (2-medial and 1-lateral K-wires)
EF: Lateral external fixation system
ESIN: Elastic stable intramedullary nails
FE: Finite element
K-wires: Kirschner wires
MDJ: Metaphyseal-diaphyseal junction
SHFs: Supracondylar humerus fractures
